# SARS-CoV-2 infection and the risk of depressive symptoms: a retrospective longitudinal study from the population-based CONSTANCES cohort

**DOI:** 10.1017/S0033291724002435

**Published:** 2024-10

**Authors:** Baptiste Pignon, Emmanuel Wiernik, Brigitte Ranque, Olivier Robineau, Fabrice Carrat, Gianluca Severi, Mathilde Touvier, Clément Gouraud, Charles Ouazana Vedrines, Victor Pitron, Nicolas Hoertel, Sofiane Kab, Sarah Tebeka, Marcel Goldberg, Marie Zins, Cédric Lemogne

**Affiliations:** 1 Université Paris Cité, Paris Saclay University, Université de Versailles Saint-Quentin-en-Yvelines, INSERM, UMS 011 « Population-based Cohorts Unit », Paris, France; 2 Service de Médecine interne, AP-HP, Hôpital européen Georges-Pompidou, Paris, France; 3 Université Paris Cité and Université Sorbonne Paris Nord, Inserm, INRAE, Center for Research in Epidemiology and StatisticS (CRESS), Paris, France; 4 Sorbonne Université, Inserm, Institut Pierre-Louis d'Epidémiologie et de Santé Publique, Paris, France; 5 EA2694, Univ Lille, Centre Hospitalier de Tourcoing, Tourcoing, France; 6 Département de santé publique, AP-HP, Hôpital Saint-Antoine, Paris, France; 7 Université Paris-Saclay, UVSQ, INSERM, CESP U1018, Gustave Roussy, Villejuif, France; 8 Department of Statistics, Computer Science, Applications ‘G. Parenti,’ University of Florence, Florence, Italy; 9 Sorbonne Paris Nord University, Inserm U1153, Inrae U1125, Cnam, Nutritional Epidemiology Research Team (EREN), Centre of Research in Epidemiology and Statistics (CRESS) – Université Paris Cité (CRESS), Bobigny, France; 10 Service de Psychiatrie de l'adulte, AP-HP, Hôpital Hôtel-Dieu, Paris, France; 11 Université Paris Cité, VIFASOM (Vigilance Fatigue Sommeil et Santé Publique), Paris, France; 12 Centre du Sommeil et de la Vigilance-Pathologie professionnelle, APHP, Hôtel-Dieu, Paris, France; 13 Université Paris Cité, INSERM U1266, Institut de Psychiatrie et Neuroscience de Paris, Paris, France; 14 Service de Psychiatrie et Addictologie, AP-HP, Hôpital Corentin-Celton, DMU Psychiatrie et Addictologie, Issy-les-Moulineaux, France

**Keywords:** COVID-19, depression, depressive symptoms, long COVID, persistent symptoms, post-COVID-19 condition

## Abstract

**Background:**

Should COVID-19 have a direct impact on the risk of depression, it would suggest specific pathways for prevention and treatment. In this retrospective population-based study, we aimed to examine the association of prior SARS-CoV-2 infection with depressive symptoms, distinguishing self-reported *v*. biologically confirmed COVID-19.

**Methods:**

32 007 participants from the SAPRIS survey nested in the French CONSTANCES cohort were included. COVID-19 was measured as followed: *ad hoc* serologic testing, self-reported PCR or serology positive test results, and self-reported COVID-19. Depressive symptoms were measured with the Center of Epidemiologic Studies-Depression Scale (CES-D). Outcomes were depressive symptoms (total CES-D score, its four dimensions, and clinically significant depressive symptoms) and exposure was prior COVID-19 (no COVID-19/self-reported unconfirmed COVID-19/biologically confirmed COVID-19).

**Results:**

In comparison to participants without COVID-19, participants with self-reported unconfirmed COVID-19 and biologically confirmed COVID-19 had higher CES-D scores (*β* for one interquartile range increase [95% CI]: 0.15 [0.08–0.22] and 0.09 [0.05–0.13], respectively) and somatic complaints dimension scores (0.15 [0.09–0.21] and 0.10 [0.07–0.13]). Only those with self-reported but unconfirmed COVID-19 had higher depressed affect dimension scores (0.08 [0.01–0.14]). Accounting for *ad hoc* serologic testing only, the CES-D score and the somatic complaints dimension were only associated with the combination of self-reported COVID-19 and negative serology test results.

**Conclusions:**

The association between COVID-19 and depressive symptoms was merely driven by somatic symptoms of depression and did not follow a gradient consistent with the hypothesis of a direct impact of SARS-CoV-2 infection on the risk of depression.

## Introduction

Since the onset of the COVID-19 pandemic, concerns have been raised about its impact on mental health (Holmes et al., 2020; Xiang et al., 2020). Early studies conducted in the general population suggested a significant and global increase in depressive and anxiety symptoms between the pre- and mid-pandemic periods (Rogers et al., 2020; Santomauro et al., 2021). Acute infectious diseases are recognized risk factors of psychiatric disorders (Köhler-Forsberg et al., 2019; Orlovska et al., 2017), particularly mood disorders (Benros et al., 2013). One of the main proposed explanations for these associations was that the immune system response may induce neuroinflammation that contributes to the emergence of psychiatric disorders (Leboyer et al., 2016; Pape, Tamouza, Leboyer, & Zipp, 2019). A similar hypothesis has been put forward for COVID-19 in particular (Bottemanne, Delaigue, & Lemogne, 2021; Han et al., 2021).

Despite evidence that SARS-CoV-2 may have an impact on brain tissues (Douaud et al., 2022; Penninx, Benros, Klein, & Vinkers, 2022), evidence from epidemiology remains elusive as to establish a direct association between SARS-CoV-2 infection and depression in the general population, with inconsistent results. Analyzing the risk of mood disorders following COVID-19 from electronic health records in different countries with different follow-up times (90 days, 6 months, 2 years), Taquet et al., found significant associations between COVID-19 and further mood disorders, in comparison to other infectious diseases (Taquet, Geddes, Husain, Luciano, & Harrison, 2021a; Taquet, Luciano, Geddes, & Harrison, 2021b; Taquet et al., 2022). In a study of the risk of mood disorders after an hospitalization for COVID-19 in France during the year 2020, in comparison to hospitalization for another reason, Decio et al. (2022) and Geoffroy et al. (2024) found crude increases of the risk of mood disorders and depression. However, these associations were no longer significant following adjustments for confounders, in particular history of mood disorders. In a prospective study of the evolution of mental health between April 2020 and 2021 in eleven longitudinal population-based studies from the United Kingdom, Thompson et al. (2022) found an association of small magnitude between COVID-19 and depressive symptoms. However, analyzing the association according to self-report and serology status, they displayed significant variations: self-reported COVID-19 without positive serology were associated with depressive symptoms, while participants with positive serology without self-reported COVID-19 had unchanged mental health (including depression) during the study period. Strikingly, those with both self-reported COVID-19 and positive serology, thus presenting with the highest probability of having been infected with SARS-CoV-2, also had unchanged mental health (including depression) during the study period. Interestingly, Davisse-Paturet et al. (2023) found similar results when examining suicidal ideation.

In addition, the critical issue of overlapping symptoms – specifically somatic symptoms – between acute infectious diseases, enduring systemic inflammation, and depression remains unaddressed in the context of COVID-19. Fatigue, cognitive impairment, sleep disorders, loss of appetite, or anhedonia, which are core symptoms of a major depressive episode, are also core symptoms of ‘sickness behavior’ (Hart, 1988). Sickness behavior is characterized by a set of behavioral changes that occur in response to an infection and helps combat the infection and prevent its spread. Importantly, sickness behavior, which is mediated by pro-inflammatory cytokines, mainly interleukins 1β and 6 (IL-1β and IL-6) and tumor necrosis factor-α (TNF-*α*), also encompasses other depressive symptoms than somatic ones, such as social withdrawal (Shattuck & Muehlenbein, 2016). In addition, persistent symptoms may impair the quality of life of many patients months after a COVID-19 episode (Ballering, van Zon, Hartman, & Rosmalen, 2022; Robineau et al., 2022). Among frequent symptoms of this ‘post-COVID-19 condition’, frequently referred to as ‘long COVID’, fatigue, poor attention/concentration, or sleep disorders may also overlap with depressive symptoms and thus lead to overestimate the association between SARS-CoV-2 infection and subsequent depression in epidemiological studies (Soriano, Murthy, Marshall, Relan, & Diaz, 2022).

This longitudinal study aimed to examine the association of SARS-CoV-2 infection with subsequent depressive symptoms in a large population-based sample. We investigated particularly the combination of self-reported COVID-19 with biological confirmation of the infection on this association, while considering various dimensions of depressive symptoms, including somatic symptoms. Regarding self-report *v.* biological confirmation, we hypothesized that we would replicate and extend the results of Thompson et al. (2022) and show stronger association of depressive symptoms with self-reported infection than with biologically-confirmed infection. We further hypothesized that much of the association between COVID-19 and depressive symptoms would be explained by a specific association with somatic symptoms of depression.

## Methods

### CONSTANCES and SAPRIS studies

The French CONSTANCES population-based cohort study received ethical approval by the institutional review board of the National Institute for Medical Research (Authorization number 910486) and included more than 200 000 volunteers aged 18–69 years at inclusion (i.e. between 2012 and 2019) who gave informed consent to be followed-up through annual questionnaires (Zins & Goldberg, 2015). Participants were selected among individuals covered by the general insurance scheme or partner health mutual societies (i.e. 85% of the French population) using a random sampling system stratified on place of residence, age, sex, occupation, and socioeconomic status. At inclusion, volunteers completed a self-administered questionnaire on lifestyle and health status and attended a Health Screening Center for a comprehensive evaluation including a physical examination and laboratory tests.

Between 2 April 2020 and 12 May 2020, a total of 63 471 volunteers of the CONSTANCES cohort responding to annual questionnaires through the internet were invited to take part in the nested SAPRIS (‘*Santé, pratiques, relations et inégalités sociales en population générale pendant la crise COVID-19*’) and SAPRIS-Sérologie (SAPRIS-SERO) surveys, which concerned specifically the impact of the COVID-19 pandemic (Carrat et al., 2021; Matta et al., 2022, 2023). The SAPRIS survey was approved by the French Institute of Health and Medical Research ethics committee, and the SAPRIS-SERO survey was approved by the Sud-Méditerranée III ethics committee. Participants were not offered any kind of incentive for participating neither in CONSTANCES nor in SAPRIS.

**Figure 1.**
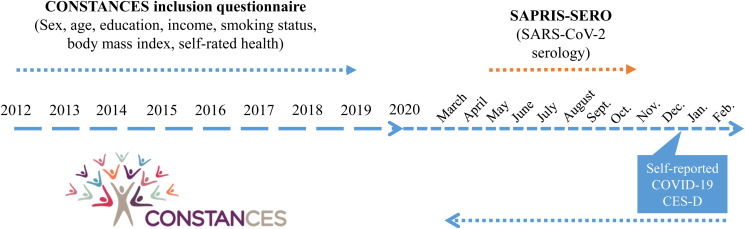
Time frame of the study based on data from CONSTANCES cohort and nested SAPRIS and SAPRIS-SERO surveys. Inclusion in the CONSTANCES cohort occurred between 2012 and 2019. Among CONSTANCES volunteers, inclusion in SAPRIS and SAPRIS-SERO surveys occurred between 2 April 2020 and 12 May 2020. SAPRIS *ad hoc* SARS-CoV-2 serological tests were conducted between May and October 2020. Self-reported COVID-19 was measured retrospectively between December 2020 and February 2021. Center of Epidemiologic Studies-Depression Scale (CES-D) was completed between December 2020 and February 2021.

The present study is a retrospective longitudinal analysis of data from the French CONSTANCES cohort and from the SAPRIS and SAPRIS-SERO surveys nested in the CONSTANCES cohort. Figure 1 shows the different steps of inclusion and data collection.

### SARS-CoV-2 infection

#### SAPRIS serology

Between May and October 2020 (i.e. a period without a wide availability of PCR tests), self-sampling dried blood spot kits were mailed to each participant, with kit material and printed instructions to mail a blood spot to a centralized biobank. Received blood spots were visually assessed, registered, punched, and stored in tubes (0.5 mL, FluidX 96-Format 2D code; Brooks Life Sciences) at −30 °C. Eluates were processed with an enzyme-linked immunosorbent assay (Euroimmun) to detect anti-SARS-CoV-2 antibodies (IgG) directed against the S1 domain of the virus spike protein. A test was considered positive for SARS-CoV-2 when the results indicated an optical density ratio of 1.1 or greater (sensitivity/specificity: 0.870/0.975). To reduce the risk of false-negative results, participants with indeterminate results (i.e. optical density ratio ≥ 0.8 and <1.1) and without declaration of an otherwise positive test were excluded.

#### Self-reported infection

Between December 2020 and February 2021, participants answered the question: ‘*Since March, do you think you have been infected with the coronavirus* (*whether or not confirmed by a physician or a test*)*?*’. Participants answered ‘*Yes*’, ‘*No*’, or ‘*I don't know*’, and participants who answered ‘*Yes*’ were additionally asked whether their infection had been confirmed, with two possible responses indicating biological confirmation: ‘*Yes, by virological or PCR test* (*based on nose swab, results provided after at least 24 h*)’, ‘*Yes, by serological test* (*based on a blood test, results provided after at least 24 h*)’.

### Assessment of depressive symptoms

Between December 2020 and February 2021, simultaneously with the assessment of self-reported infection, depressive symptoms were assessed using the self-administered Center of Epidemiologic Studies-Depression Scale (CES-D) (Morin et al., 2011). This 20-item scale assesses the frequency of depressive symptoms during the previous week. Reponses range from 0 (mostly never) to 3 (most of the time), resulting in a total score ranging from 0 to 60. According to the validated cutoff of the French version, ‘clinically significant depressive symptoms’ were defined as having a total score ≥ 19 (sensitivity/specificity for major depression: 0.85/0.86). Based on the dimensional structure of the French version of the CES-D, the four following dimensions of the CES-D were also assessed: depressed affect (7 items, scores between 0 and 21), positive affect (4 items, scores between 0 and 12), somatic complaints (7 items, scores between 0 and 21), and disturbed interpersonal relationships (2 items, scores between 0 and 6).

### Adjustment variables

Sex and age were obtained from the national personal identification directory. Educational level, household income, and current tobacco smoking status were self-reported at inclusion in the CONSTANCES cohort. Body mass index (BMI) was categorized in four categories (<18.5 kg/m^2^: underweighted, 18.5–25: normal, 25–30: overweighted, >30: obese) from weight and height measured in Health Screening Centers at inclusion. Self-rated health (‘*How do you rate your overall health?*’) was assessed according to a score between 1 to 8 (1: ‘*very bad*’, 8: ‘*very good*’) at the same time as CES-D.

### Statistical analyses

Six dependent variables were considered for depressive symptoms, including five continuous variables (i.e. total CES-D score and its four dimensions) and one binary variable (i.e. clinically significant depressive symptoms defined by a total CES-D score ≥ 19). For the total CES-D score, person mean imputation was performed if 2 items or less were missing (Hawthorne, Hawthorne, & Elliott, 2005). If more than 2 items were missing, the subjects were excluded from the current analyses.

Unadjusted analyses first considered COVID-19 status as a 3-category variable: no COVID-19/self-reported unconfirmed COVID-19 (i.e. neither a positive SAPRIS serology nor self-reported serology or PCR test results)/biologically confirmed COVID-19, self-reported or not. Pairwise mean comparisons were performed with *t*-Student tests and χ^2^ test, for continuous and binary dependent variables, respectively.

Adjusted linear and/or binary logistic regression models were then computed for continuous and/or binary dependent variables previously associated with the 3-category COVID-19 status variable in unadjusted analyses. All *a priori* selected covariates were included in the models (i.e. sex, age, educational level, household income, smoking status, BMI, and self-rated health). The continuous dependent variables (i.e. CES-D total score and its four dimensions) were divided by their interquartile range (IQR), to yield interpretable regression coefficients and 95% confidence intervals (CIs).

Finally, in order to replicate the findings by Thompson et al. (2022), these adjusted analyses were repeated with COVID-19 status as a 4-category variable based on self-report and SAPRIS serology only: no self-reported COVID-19 and SAPRIS serology negative test results/self-reported COVID-19 and SAPRIS serology negative test results/no self-reported COVID-19 and SAPRIS serology positive test results/self-reported COVID-19 and SAPRIS serology positive test results. For these analyses, participants with SAPRIS serology negative test results who later reported positive PCR or serology test results were excluded.

## Results

### Participants

Among the 63 541 volunteers from CONSTANCES cohort who were included in the SAPRIS survey, 28 704 participants were not included in this study because they were not included in SAPRIS-SERO study and 786 because they had indeterminate SAPRIS-SERO serology test results without self-reported serology or PCR positive test results. Of the 34 041 remaining participants, 2034 had unavailable data concerning self-reported COVID-19. A total of 32 007 participants were thus included in the present study. Among these participants, 925 reported positive PCR or serology test results after negative SAPRIS serology and were not included in the analyses based on the 4-category COVID-19 status variable.

The characteristics of the participants (total sample, samples with and without self-reported and/or biologically confirmed COVID-19) are displayed in Table 1. Women were overrepresented (51.6%). Overall mean age was 52.5 years (standard deviation (s.d.) = 13.3). While 28 659 participants (89.5%) did not have any self-reported or biologically confirmed COVID-19 episode, 666 (2.1%) self-reported a COVID-19 episode without biological confirmation, and 2682 (8.4%) had a biologically confirmed COVID-19.

**Table 1.** Characteristics of the participants according to COVID-19 status

	Total sample (*N* = 32 007)		COVID-19 status					
			No COVID-19 (*N* = 28 659)		Self-reported unconfirmed COVID-19 (*N* = 666)		Biologically confirmed COVID-19 (*N* = 2682)	
	Mean	s.d.	Mean	s.d.	Mean	s.d.	Mean	s.d.
CES-D total score	14.4	5.8	14.3	5.8	16.0	6.3	15.3	5.9
CES-D depressed affect dimension	2.5	3.3	2.5	3.3	3.2	3.9	2.8	3.4
CES-D positive affect dimension	7.9	3.0	7.9	3.0	7.9	2.9	7.9	3.0
CES-D somatic complaints dimension	3.6	3.4	3.5	3.3	4.6	3.8	4.2	3.6
CES-D interpersonal relationships dimension	3.6	0.8	3.5	0.8	4.6	0.8	4.2	0.8
Age	52.7	13.2	53.4	13.2	46.4	12.5	47.3	12.7
	*N*	%	*N*	%	*N*	%	*N*	%
Clinically significant depressive symptoms^a^	5870	18.3	5080	15.7	180	27.0	610	22.7
Female sex	16 480	51.4	14 678	51.2	384	57.7	1418	52.3
Educational level^b^								
1	4077	12.7	3800	13.3	44	6.6	233	8.7
2	4312	13.5	3933	13.7	80	12.0	299	11.1
3	8873	27.7	7934	27.7	177	26.6	762	28.4
4	3501	10.9	3126	10.9	69	10.4	306	11.4
5	10 896	34.0	9552	33.3	290	43.5	1054	39.3
Monthly household income (€)								
<2100	3600	11.2	3211	11.2	86	12.9	303	11.3
2100-2800	3817	11.9	3451	12.0	69	10.4	297	11.1
2800-4200	10 204	31.9	3740	13.1	202	30.3	862	32.1
>4200	13 022	40.7	11 644	40.6	271	40.7	1107	41.3
Smokers^c^	3861	12.1	3463	12.1	93	14.0	305	11.4
Body mass index								
<18.5	18 457	57.7	16 468	57.5	423	63.5	1566	58.4
18.5-25	830	2.6	726	2.5	23	3.5	81	3.0
25-30	9337	29.2	8403	29.3	173	26.0	761	28.4
>30	2961	9.3	2681	9.4	41	6.2	239	8.9
Self-rated health^d^								
1	57	0.2	57	0.2	0	0.0	0	0.0
2	332	1.0	288	1.0	8	1.2	36	1.3
3	649	2.0	565	2.0	21	3.2	63	2.3
4	858	2.7	739	2.6	34	5.1	85	3.2
5	1673	5.2	1471	5.1	40	6.0	162	6.0
6	5871	18.3	5241	18.3	129	19.4	501	18.7
7	16 386	51.2	14 753	51.5	318	47.7	1315	49.0
8	5948	18.6	5378	18.8	112	16.8	458	17.1

Abbreviations: s.d., standard-deviation; CES-D, Center of Epidemiologic Studies-Depression scale.

a According to CES-D binarized score: participants with a score ≥ 19 are considered with clinically significant depressive symptoms.

b Educational level: 1 = without diploma, without high school diploma, or with certificate of vocational aptitude or vocational studies; 2 = high school diploma or equivalent; 3 = 2 or 3 years of post-secondary education, 4 = 4 years of post-secondary education; 5 = 5 years or more of post-secondary education.

c At the inclusion in CONSTANCES cohort.

d Self-rated health 1: ‘*very bad*’ to 8: ‘*very good*’.

### Unadjusted analyses

Pairwise mean comparisons revealed an increasing gradient of the mean scores for the CES-D total, depressed affect and somatic complaints dimensions, across the three categories as follows: first, no COVID-19, then biologically confirmed COVID-19, and third self-reported unconfirmed COVID-19 (Table 2). The same gradient was found for the prevalence of ‘clinically significant depressive symptoms’, but not for the positive affect or disturbed interpersonal relationships CES-D dimension scores.

**Table 2.** Comparisons of mean^a^ of CES-D scores or proportion of clinically significant depressive symptoms^b^ according to the 3-category COVID-19 status

	Means or proportions			Comparisons of means or proportions		
	No COVID-19 (*N* = 28 659)	Self-reported unconfirmed COVID-19 (*N* = 666)	Biologically confirmed COVID-19 (*N* = 2682)	No *v*. biologically confirmed COVID-19	Self-reported unconfirmed *v*. no COVID-19	Biologically confirmed *v*. self-reported unconfirmed COVID-19
Mean CES-D total score	14.3	16.0	15.3	*t* = −8.83, df = 30 271, *p* < 0.001	*t* = −7.62, df = 28 377, *p* < 0.001	*t* = 2.62, df = 3198, *p* = 0.009
Mean CES-D depressed affect dimension score	2.5	3.2	2.7	*t* = −3.97, df = 30 618, *p* < 0.001	*t* = −5.19, df = 28 694, *p* < 0.001	*t* = 2.64, df = 3232, *p* = 0.008
Mean CES-D positive affect dimension	7.2	7.9	7.9	*t* = −0.48, df = 30 526, *p* = 0.633	*t* = 0.15, df = 28 605, *p* = 0.881	*t* = −0.37, df = 3233, *p* = 0.715
Mean CES-D somatic complaints dimension	3.5	4.6	4.2	*t* = −9.99, df = 30 675, *p* < 0.001	*t* = −8.14, df = 28 744, *p* < 0.001	*t* = 2.43, df = 3239, *p* = 0.015
Mean CES-D disturbed interpersonal relationships dimension score	0.3	0.4	0.4	*t* = −2.05, df = 30 719, *p* = 0.040	*t* = −1.28, df = 28 783, *p* = 0.200	*t* = 0.19, df = 3248, *p* = 0.849
Number and proportion of participants with clinically significant depressive symptoms^c^	*N* = 5080, 17.3%	*N* = 180, 27.0%	*N* = 610, 22.7%	χ^2^ = 48.04, *p* < 0.001	χ^2^ = 35.49, *p* < 0.001	χ^2^ = 820.12, *p* < 0.001

Abbreviations: CES-D, Center of Epidemiologic Studies-Depression Scale; df, degrees of freedom.

a Using *t*-Student tests.

b Using χ^2^ tests.

c According to CES-D binarized score: participants with a score ≥ 19 are considered with clinically significant depressive symptoms.

### Adjusted analyses

In unadjusted analyses, CES-D total score (Table 3), as well as depressed affect and somatic complaints dimension scores, were the lowest in non-infected participants, intermediate in participants with biologically confirmed COVID-19 and highest in participants with self-reported unconfirmed COVID-19. Adjusted analyses were consistent for total score and somatic complaints dimension. Only participants with self-reported unconfirmed COVID-19 had higher depressed affect dimension score, in comparison to participants without prior infection. Concerning depressed affect dimension score, in adjusted analyses, no significant variations were observed. The positive affect and the disturbed interpersonal relationships dimensions score were not associated with the COVID-19 status. Clinically significant depressive symptoms were more frequent in participants with biologically confirmed COVID-19 and, with a greater effect size, in those with self-reported unconfirmed COVID-19 in both unadjusted and adjusted analyses.

**Table 3.** Analyses of the association of the 3-category COVID-19 status and CES-D scores or clinically significant depressive symptoms

	Unadjusted analyses			Multivariable analyses^a^		
	*β*	95% CI−	95% CI+	*β*	95% CI−	95% CI+
CES-D total score^b^						
No COVID-19	*ref.*	*ref.*	*ref.*	*ref.*	*ref.*	*ref.*
Self-reported unconfirmed COVID-19	0.29***	0.22	0.36	0.15***	0.08	0.22
Biologically confirmed COVID-19	0.18***	0.14	0.21	0.09***	0.05	0.13
Depressed affect CES-D dimension^b^						
No COVID-19	*ref.*	*ref.*	*ref.*	*ref.*	*ref.*	*ref.*
Self-reported unconfirmed COVID-19	0.17***	0.11	0.23	0.08*	0.01	0.14
Biologically confirmed COVID-19	0.07***	0.03	0.10	0.01	−0.02	0.04
Positive affect CES-D dimension^b^						
No COVID-19	*ref.*	*ref.*	*ref.*	*ref.*	*ref.*	*ref.*
Self-reported unconfirmed COVID-19	0.00	−0.06	0.05	0.00	−0.06	0.06
Biologically confirmed COVID-19	0.01	−0.02	0.04	0.02	−0.01	0.05
Somatic complaints CES-D dimension^b^						
Absence of COVID-19	*ref.*	*ref.*	*ref.*	*ref.*	*ref.*	*ref.*
Self-reported unconfirmed COVID-19	0.27***	0.20	0.33	0.15***	0.09	0.21
Biologically confirmed COVID-19	0.17***	0.14	0.21	0.10***	0.07	0.13
Disturbed interpersonal relation CES-D dimension^b^						
Absence of COVID-19	*ref.*	*ref.*	*ref.*	*ref.*	*ref.*	*ref.*
Self-reported unconfirmed COVID-19	0.04	−0.02	0.10	−0.01	−0.07	0.05
Biologically confirmed COVID-19	0.03*	0.00	0.06	0.00	−0.03	0.04
	OR	95% CI−	95% CI+	OR	95% CI−	95% CI+
Clinically significant depressive symptoms (i.e. CES-D total score ≥ 19)						
Absence of COVID-19	*ref.*	*ref.*	*ref.*	*ref.*	*ref.*	*ref.*
Self-reported unconfirmed COVID-19	1.10***	1.06	1.13	1.05**	1.02	1.08
Biologically confirmed COVID-19	1.05***	1.04	1.07	1.03***	1.02	1.05

Abbreviations: CI−, lower confidence limit; CI+, upper confidence limit; IQR, interquartile range; OR, odds ratio; ref., reference value.

a Adjusted on sex, age, educational level, household income, smoking status, BMI, and self-rated health.

b CES-D scores are divided par the IQR.

*p value < 0.05, **p value < 0.01, ***p value < 0.001.

Among the adjustment factors, in the different models, female sex, higher educational level, tobacco smoking, younger age, lower household income, obese status, and self-rated health were associated with greater depressive symptoms (online Supplementary Table S1).

Finally, regarding SAPRIS serology test results specifically, 323 participants did not report having been infected with SARS-CoV-2 while they had positive test results, 822 reported COVID-19 while they had negative test results, and 632 reported COVID-19 with positive test results, while 28 659 did not report COVID-19 with negative test results. In comparison to the absence of COVID-19 history (according to both self-report and SAPRIS serology test results), only participants with self-reported COVID-19 with negative SAPRIS serology test results had higher CES-D total scores (Table 4, see online Supplementary Table S2 for details concerning adjustment factors). Participants with self-reported COVID-19 had higher depressed affect and somatic complaints dimension scores, especially those with negative SAPRIS serology test results. Positive affect and disturbed interpersonal relationship dimension scores did not significantly vary according to the four-category COVID-19 variable. The proportion of ‘clinically significant depressive symptoms’ was higher among participants who self-reported COVID-19, whatever the SAPRIS serology test results.

**Table 4.** Adjusted^a^ association between CES-D scores, clinically significant depressive symptoms, and the 4-category COVID-19 status

		*β*	95% CI−	95% CI+
CES-D total score^b^				
Negative SAPRIS serology test results	No self-reported COVID-19	*Ref.*	*Ref.*	*Ref.*
	Self-reported COVID-19	0.14***	0.07	0.21
Positive SAPRIS serology test result	No self-reported COVID-19	0.06	−0.05	0.16
	Self-reported COVID-19	0.06	−0.02	0.13
Depressed affect CES-D dimension^b^				
Negative SAPRIS serology test result	No self-reported COVID-19	*Ref.*	*Ref.*	*Ref.*
	Self-reported COVID-19	0.08**	0.02	0.13
Positive SAPRIS serology test result	No self-reported COVID-19	0.00	−0.09	0.09
	Self-reported COVID-19	0.00	−0.06	0.07
Positive affect CES-D dimension^b^				
Negative SAPRIS serology test result	No self-reported COVID-19	*Ref.*	*Ref.*	*Ref.*
	Self-reported COVID-19	−0.01	−0.07	0.04
Positive SAPRIS serology test result	No self-reported COVID-19	0.04	−0.04	0.12
	Self-reported COVID-19	0.01	−0.05	0.06
Somatic complaints CES-D dimension^b^				
Negative SAPRIS serology test result	No self-reported COVID-19	*Ref.*	*Ref.*	*Ref.*
	Self-reported COVID-19	0.15***	0.09	0.20
Positive SAPRIS serology test result	No self-reported COVID-19	0.03	−0.06	0.12
	Self-reported COVID-19	0.06	0.00	0.12
Disturbed interpersonal relationships CES-D dimension^b^				
Negative SAPRIS serology test result	No self-reported COVID-19	*Ref.*	*Ref.*	*Ref.*
	Self-reported COVID-19	0.00	−0.05	0.06
Positive SAPRIS serology test result	No self-reported COVID-19	0.01	−0.08	0.10
	Self-reported COVID-19	0.02	−0.04	0.08
		OR	95% CI−	95% CI+
Clinically significant depressive symptoms (i.e. CES-D total score ≥ 19)				
Negative SAPRIS serology negative test result	No self-reported COVID-19	*Ref.*	*Ref.*	*Ref.*
	Self-reported COVID-19	1.05**	1.02	1.08
Positive SAPRIS serology positive test result	No self-reported COVID-19	1.02	0.98	1.07
	Self-reported COVID-19	1.03*	1.00	1.07

Abbreviations: CI−, lower confidence limit; CI+, upper confidence limit; IQR, interquartile range; OR, odds ratio; ref., reference value.

a Adjusted on sex, age, educational level, household income, smoking status, BMI, and self-rated health.

b CES-D scores are divided par the IQR.

*p value < 0.05, **p value < 0.01, ***p value < 0.001.

## Discussion

This longitudinal population-based study aimed to examine the relationships between a history of SARS-CoV-2 infection and depressive symptoms according to self-report *v.* biological confirmation and across different dimensions of depressive symptoms. Taking into account both PCR and serology test results, participants with self-reported COVID-19 without biological confirmation and those with biologically confirmed COVID-19 had both higher levels of subsequent depressive symptoms. A gradient of depressive symptoms was observed from participants with no history of COVID-19 (lowest) to those with biologically confirmed COVID-19 (intermediate), and those with self-reported unconfirmed COVID-19 (highest). In addition, among the four CES-D specific dimensions, only the somatic complaints dimension was associated with a biologically confirmed history of COVID-19. The depressed affect dimension was associated with self-reported unconfirmed COVID-19 only. Altogether, these results are not consistent with the hypothesis of a direct impact of SARS-CoV-2 infection on the risk of depression.

This study has several strengths, including its population-based nature, the fairly large sample, and the longitudinal design. The dimensional assessment of depressive symptoms, with both total and specific dimensions, is another major strength of this study. Moreover, participants' SARS-CoV-2 infection status was measured with different methods (*ad hoc* serologic testing and/or self-reported positive PCR/serology test results). The time frame of the study makes the results relatively independent of the effects of viral variants that followed the wild type after the second wave of the pandemic.

This study has also limitations. First, the date of SARS-CoV-2 infection could not be precisely determined. Second, misclassification may have occurred regarding COVID-19 status. For instance, false positives may result from the combination of serologic testing low specificity with low prevalence of prior SARS-CoV-2 infection at the time of the study, especially in those not reporting COVID-19, as well as from low specificity of self-report, since other viruses may cause similar symptoms. On the other hand, false negatives may result from low sensitivity of PCR test results (encompassing participants who did not had PCR testing at the time of infection), especially in those reporting COVID-19, as well as from low sensitivity of self-report since SARS-CoV-2 infection could be asymptomatic in many cases (Sah et al., 2021). Other limitations included the reliance on the CES-D, which is a well-validated scale in population-based studies but does not allow diagnosing major depression. Finally, of note, our sample could not be considered as representative of the general population, and the rate of SARS-CoV-2 infection in our sample was slightly lower than in the general population at the same time (10.5% in our sample *v.* 13.7% in the general population) (Salje et al., 2020; Santé Publique France, 2023).

The fact that the somatic complaints dimension was, among the four considered depressive symptom dimensions, the most strongly associated with prior COVID-19, suggests that this association may be partially explained by overlapping somatic persistent symptoms following SARS-CoV-2 infection, such as those typically observed in long COVID (e.g. fatigue, poor attention/concentration, sleep disorders) (Pignon et al., 2024). For instance, the sickness behavior associated with circulating pro-inflammatory cytokines, which are under intense scrutiny in long COVID (Klein et al., 2023), also includes typical depressive symptoms such as fatigue, poor attention/concentration, social withdrawal, sleep disorders, loss of appetite, or anhedonia (Hart, 1988; Shattuck & Muehlenbein, 2016). However, the results of immunological studies in long COVID are not consistent regarding the nature of circulating pro-inflammatory cytokines or dysregulated immune cells (Patterson et al., 2021; Phetsouphanh et al., 2022; Schultheiß et al., 2023). Besides, it is unclear whether the observed low grade inflammation relates to prior SARS-CoV-2 infection irrespective of the presence of persistent symptoms (Lund Berven et al., 2022; Sommen et al., 2022), long COVID (Klein et al., 2023), or associated features such as low physical activity (Gleeson et al., 2011) or sleep disturbances (Irwin, Olmstead, & Carroll, 2016). Furthermore, the results of the present study are indeed not consistent with a direct biological effect of SARS-CoV-2 infection on the risk of depression.

According to the hypothesis of a direct link between SARS-CoV-2 infection and depression, the gradient of depressive symptoms across the different COVID-19 status categories would have followed the probability of prior SARS-CoV-2 infection, which is arguably the lowest in participants who did not report COVID-19 (either suspected or biologically confirmed) and had negative SAPRIS serology test results, and the highest in those with biologically confirmed COVID-19, with intermediate probability in those with self-reported, yet unconfirmed COVID-19. However, depressive symptoms were higher among subjects with self-reported unconfirmed COVID-19 than among subjects with biologically confirmed COVID-19. One might argue than among those with biologically confirmed COVID-19, false positives among those who did not report COVID-19 but had a positive SAPRIS serology test results could have diluted the effect. However, when splitting our population into four groups according to self-report and SAPRIS serology test results, only participants with self-reported COVID-19 but negative SAPRIS serology test results displayed higher levels of depressive symptoms than those without COVID-19, while those with self-reported COVID-19 and positive SAPRIS serology test results did not, despite an undoubtedly higher probability of prior SARS-CoV-2 infection. These results are consistent with Thompson et al. (2022) and Davisse-Paturet et al. (2023) studies.

One should keep in mind the clinical heterogeneity of the sample and the fact that few participants with SARS-CoV-2 infection and severe COVID-19 may have experienced encephalopathy (Liotta et al., 2020). These patients are at higher risk of subsequent psychiatric disorders, including depression (Granerod et al., 2017; Varatharaj et al., 2020).

Overall, our results suggest that depressive episodes occurring after COVID-19 may not be attributable to a direct impact of SARS-CoV-2. Regarding clinical implications, they suggest that post-COVID-19 depressive episodes should be treated following current international guidelines for depressive disorder, without being misguided by biologically plausible but epidemiologically unsubstantiated hypotheses. Regarding implications for research, these results suggest that the exclusion of depressive symptoms from the definition of the post-COVID-19 condition might be worth considering. Informed by accumulating evidence from epidemiological studies (Tebeka et al., 2023), such exclusion would diminish the heterogeneity of this condition, and help the search for potential biomarkers and/or adequate treatments.

## Supporting information

Pignon et al. supplementary material 1 Pignon et al. supplementary material

Pignon et al. supplementary material 2 Pignon et al. supplementary material

## Supplementary material

The supplementary material for this article can be found at https://doi.org/10.1017/S0033291724002435.
