# Supplementary material for: SARS-CoV-2 infection and the risk of depressive symptoms: a retrospective longitudinal study from the population-based CONSTANCES cohort
Source: Psychol Med. 2024 Oct 14;54(14):3939–48. doi: 10.1017/S0033291724002435 (PMC11578902; doi:10.1017/S0033291724002435)
Supplement: Pignon et al. supplementary material 2 — Pignon et al. supplementary material [file S0033291724002435sup002.docx]

| **Supplementary Table 2. Adjusted association between CES-D scores, clinically significant depressive symptoms, and the 4-category COVID-19 status** | | | | |
| --- | --- | --- | --- | --- |
|  | | ß | 95% CI- | 95 % CI+ |
| *CES-D total score^1^* | | | | |
| Negative SAPRIS serology test results | No self-reported COVID-19 | *ref.* | *ref.* | *ref.* |
|  | Self-reported COVID-19 | 0.14*** | 0.07 | 0.21 |
| Positive SAPRIS serology test results | No self-reported COVID-19 | 0.06 | -0.05 | 0.16 |
|  | Self-reported COVID-19 | 0.06 | -0.02 | 0.13 |
| Female sex | | 0.32*** | 0.30 | 0.34 |
| Age (per 10 years) | | -0.08*** | -0.09 | -0.07 |
| Educational level | | 0.07*** | 0.06 | 0.08 |
| Household income | | -0.05*** | -0.06 | -0.04 |
| Smokers | | 0.08*** | 0.05 | 0.11 |
| Body mass index <18.5 | | 0.06 | -0.01 | 0.13 |
| 18.5-25 | | *ref.* | *ref.* | *ref.* |
| 25-30 | | -0.01 | -0.04 | 0.01 |
| >30 | | 0.04* | 0.01 | 0.08 |
| Self-rated health | | 0.22*** | 0.21 | 0.23 |
| *Depressed affect CES-D dimension^1^* | | | | |
| Negative SAPRIS serology test results | No self-reported COVID-19 | *ref.* | *ref.* | *ref.* |
|  | Self-reported COVID-19 | 0.08** | 0.02 | 0.13 |
| Positive SAPRIS serology test results | No self-reported COVID-19 | 0.00 | -0.09 | 0.09 |
|  | Self-reported COVID-19 | 0.00 | -0.06 | 0.07 |
| Female sex | | 0.27*** | 0.25 | 0.28 |
| Age (per 10 years) | | -0.05*** | -0.05 | -0.04 |
| Educational level | | 0.03*** | 0.03 | 0.04 |
| Household income | | -0.08*** | -0.09 | -0.07 |
| Smokers | | 0.09*** | 0.06 | 0.11 |
| Body mass index < 18.5 | | 0.07* | 0.02 | 0.13 |
| 18.5-25 | | *ref.* | *ref.* | *ref.* |
| 25-30 | | -0.02* | -0.05 | 0.00 |
| >30 | | 0.01 | -0.03 | 0.04 |
| Self-rated health | | 0.21*** | 0.21 | 0.22 |
| *Positive affect CES-D dimension^1^* | | | | |
| Negative SAPRIS serology test results | No self-reported COVID-19 | *ref.* | *ref.* | *ref.* |
|  | Self-reported COVID-19 | -0.01 | -0.07 | 0.04 |
| Positive SAPRIS serology test results | No self-reported COVID-19 | 0.04 | -0.04 | 0.12 |
|  | Self-reported COVID-19 | 0.01 | -0.05 | 0.06 |
| Female sex | | -0.06*** | -0.08 | -0.04 |
| Age (per 10 years) | | 0.00 | -0.01 | 0.00 |
| Educational level | | 0.02*** | 0.02 | 0.03 |
| Household income | | 0.06*** | 0.05 | 0.07 |
| Smokers | | -0.06*** | -0.08 | -0.03 |
| Body mass index <18.5 | | -0.04 | -0.09 | -0.02 |
| 18.5-25 | | *ref.* | *ref.* | *ref.* |
| 25-30 | | 0.02* | 0.00 | 0.04 |
| >30 | | 0.02 | -0.01 | 0.05 |
| Self-rated health | | -0.18*** | -0.18 | -0.17 |
| *Somatic complaints CES-D dimension^1^* | | | | |
| Negative SAPRIS serology test results | No self-reported COVID-19 | *ref.* | *ref.* | *ref.* |
|  | Self-reported COVID-19 | 0.15*** | 0.09 | 0.20 |
| Positive SAPRIS serology = test results | No self-reported COVID-19 | 0.03 | -0.06 | 0.12 |
|  | Self-reported COVID-19 | 0.06 | 0.00 | 0.12 |
| Female sex | | 0.25*** | 0.23 | 0.27 |
| Age (per 10 years) | | -0.06*** | -0.07 | -0.05 |
| Educational level | | 0.04*** | 0.03 | 0.05 |
| Household income | | -0.04*** | -0.05 | -0.04 |
| Smokers | | 0.09*** | 0.06 | 0.12 |
| Body mass index <18.5 | | 0.05 | -0.01 | 0.10 |
| 18.5-25 | | *ref.* | *ref.* | *ref.* |
| 25-30 | | -0.02 | -0.04 | 0.00 |
| >30 | | 0.03 | -0.01 | 0.06 |
| Self-rated health | | 0.26*** | 0.25 | 0.27 |
| *Disturbed interpersonal relation CES-D dimension^1^* | | | | |
| Negative SAPRIS serology test results | No self-reported COVID-19 | *ref.* | *ref.* | *ref.* |
|  | Self-reported COVID-19 | 0.00 | -0.05 | 0.06 |
| Positive SAPRIS serology test results | No self-reported COVID-19 | 0.01 | -0.08 | 0.10 |
|  | Self-reported COVID-19 | 0.02 | -0.04 | 0.08 |
| Female sex | | 0.10*** | 0.08 | 0.12 |
| Age (per 10 years) | | -0.04*** | -0.04 | -0.03 |
| Educational level | | 0.01* | 0.00 | 0.02 |
| Household income | | -0.04*** | -0.05 | -0.03 |
| Smokers | | 0.02 | 0.00 | 0.05 |
| Body mass index <18.5 | | 0.04 | -0.02 | 0.10 |
| 18.5-25 | | *ref.* | *ref.* | *ref.* |
| 25-30 | | 0.01 | -0.01 | 0.03 |
| >30 | | 0.05** | 0.02 | 0.08 |
| Self-rated health | | 0.10*** | 0.09 | 0.11 |
|  | | OR | 95% CI- | 95% CI+ |
| *Clinically significant depressive symptoms (*i.e.*, CES-D total score ≥19)* | | | | |
| Negative SAPRIS serology test results | No self-reported COVID-19 | *ref.* | *ref.* | *ref.* |
|  | Self-reported COVID-19 | 1.05** | 1.02 | 1.08 |
| Positive SAPRIS serology test results | No self-reported COVID-19 | 1.02 | 0.98 | 1.07 |
|  | Self-reported COVID-19 | 1.03* | 1.00 | 1.07 |
| Female sex | | 1.10*** | 1.09 | 1.11 |
| Age (per 10 years) | | 0.98*** | 0.98 | 0.98 |
| Educational level | | 1.02*** | 1.01 | 1.02 |
| Household income | | 0.88*** | 0.97 | 0.98 |
| Smokers | | 1.03*** | 1.01 | 1.04 |
| Body mass index <18.5 | | 1.02 | 0.99 | 1.05 |
| 18.5-25 | | *ref.* | *ref.* | *ref.* |
| 25-30 | | 1.00 | 0.99 | 1.01 |
| >30 | | 1.02* | 1.00 | 1.04 |
| Self-rated health | | 1.08*** | 1.08 | 1.08 |
| Legends:  ^1^CES-D scores are divided par the IQR.  * p-value < 0.05, ** p-value < 0.01, *** p-value < 0.001.  Abbreviations: CI- = lower confidence limit, CI+ = upper confidence limit, IQR = interquartile range, OR = odds ratio, ref. = reference value. | | | | |
